# An In Vitro Study for the Role of Schizophrenia-Related Potential miRNAs in the Regulation of *COMT* Gene

**DOI:** 10.1007/s12035-024-04070-2

**Published:** 2024-03-01

**Authors:** Onur Tonk, Pervin Elvan Tokgun, Özge Sarıca Yılmaz, Onur Tokgun, Kubilay Inci, Büşra Çelikkaya, Nuray Altintas

**Affiliations:** 1 Faculty of Medicine, Department of Medical Biology, Celal University, Manisa, Turkey; 2 Faculty of Medicine, Department of Medical Genetics, Pamukkale University, Kınıklı, Denizli, Turkey; 3 Department of Cancer Molecular Biology, Institute of Health Sciences, Pamukkale University, Denizli, Turkey

**Keywords:** Schizophrenia, COMT gene, miR-30a-5p, miR-30e-5p, miR-34a-5p

## Abstract

This study aimed to analyze the possible association of miR-30a-5p, miR-30e-5p, and miR-34a-5p identified as potential candidate miRNAs in schizophrenia, with the COMT gene. Candidate miRNAs were obtained from the TargetScan database. The SH-SY5Y human neuroblastoma cell line was used as a cellular model for schizophrenia. miR-30a-5p, miR-30e-5p, and miR-34a-5p mimics were transfected into the SH-SY5Y cell line. Total RNA was isolated from transfected cells and RNA-IP samples and reverse transcripted for miRNA and mRNA analysis. RT-qPCR and western blot were performed to observe changes in expression levels of *COMT*. RNA-ımmunoprecipitation was performed to determine RNA–protein interactions after mimic transfection. In the study, it was observed that COMT gene expression levels decreased significantly after miR-30a-5p and miR-34a-5p expressions, whereas increased significantly as a result of miR-30e-5p transfection. RNA-IP data have shown that the amount of COMT pulled down by Ago2 was increased after miR-30a-5p and miR-34a-5p transfections. RNA-IP results revealed that miR-30a-5p and miR-34a-5p are direct targets for the *COMT* gene.

## Introduction

Schizophrenia (SCZ) is a hereditary (approximately 80%) and chronic neurodevelopmental brain disease with a genetic and neurobiological history resulting in premature death and a high prevalence of treatment resistance [1, 2]. Although schizophrenia is the most devastating psychiatric disease due to its early onset and chronicity, its pathomechanism is quite complex [3–6]. Schizophrenia affects approximately 0.5 to 0.7% of the human population [6, 7] and affects between four and seven per 1000 people worldwide [8, 9]. Due to its genomic location and its function in dopamine catabolism, COMT is considered to be a strong candidate gene that has received the most attention for schizophrenia and is promising for treatment response [10, 11]. COMT is an important enzyme that degrades catecholamines, including dopamine, and is one of the key factors involved in the regulation of dopamine levels [12–14]. Epigenetic modifications, including non-coding RNAs, play a role in many diseases such as neuropsychiatric disorders. Schizophrenia and other major psychiatric and neurodevelopmental disorders are associated with abnormalities in multiple epigenetic mechanisms [15]. miRNAs are widely distributed in different organisms and play a role in almost all life processes [16]. Through multiple mechanisms affecting transcription and translation, miRNAs can affect the expression of gene groups essential for development and lifetime cellular functioning [17]. miRNAs regulate many cell signaling pathways, can affect the physiological functioning of cells, and may, therefore, play a role in the development of schizophrenia [18]. As miRNAs are predominantly regulated transcriptionally and are greatly influenced by alterations in the biological pathway, miRNA abnormalities or mutations within the cell may cause neurological disorders, such as the pathophysiological changes observed in schizophrenia [19]. Functionally, miRNAs regulate gene expression by binding to the 3′ UTRs of mRNAs. In this way, it can inhibit the conversion of mRNA to protein due to steric inhibition of the protein synthesis mechanism or target mRNA for enzymatic degradation [20]. Mature miRNA can bind to target mRNA transcripts. As a result, it causes transcriptional repression or degradation of target mRNAs. Each miRNA can simultaneously affect the expression of hundreds of genes and synchronize multiple components of independent signaling pathways [17, 19, 21–23]. miRNAs act as a post-transcriptional gene regulator via RISC. It strengthens the interaction between the miRNA sequence and the target mRNA, forming a triple miRNA:AGO:mRNA complex. Argonaute (AGO) acts as the main protein in this regulatory complex. This complex results in a suppression of gene expression through mRNA cleavage or translational repression. The initial inhibition of protein synthesis through reduced translation is a result of the miRNA binding to its target. This is followed by degradation of the mRNA [24, 25]. Approximately 70% of human miRNAs are expressed in the nervous system, where they play various roles in regulating neural structure and function. Studies have shown that they are also involved in the development of neuropsychiatric disorders and that abnormal expression of them can be used as potential biomarkers for treatment [16].

Variations in miRNA target genes may also play a role in the development of schizophrenia, particularly in the biological pathways of miRNA [19]. Although the causes of schizophrenia are still unclear, miRNAs are fully expressed in the brain tissue of patients with schizophrenia [26].

In short, considerable research has been carried out on the functional significance of miRNA regulatory networks in neural development and brain function. Recent studies have shown that these networks play an important role in SCZ, suggesting that miRNAs may be used as potential biomarkers and targets for therapeutic intervention [27]. Studies have reported that the COMT gene, which metabolizes dopamine, epinephrine, and norepinephrine, is associated with SCZ. COMT transfers a methyl group to catecholamine due to the breakdown of neurotransmitters including dopamine, adrenaline, and noradrenaline. Studies have also shown that alterations in dopamine signaling and structural cortical maturation are associated with a genetic predisposition to SCZ.

COMT Val158Met is the genotype that has been most widely studied in psychosis. However, the association of the functional SNPs with the phenotype of schizophrenia is still unclear. Some studies suggest that patients who carry the COMT Val allele tend to have an increased risk of psychotic disorders compared to ones who carry the Met allele [28]; on the other hand, some studies have reported no association [29, 30]. Although genetic and developmental factors are generally thought to play a critical role in the pathogenesis of schizophrenia, single-nucleotide polymorphisms (SNPs) have also been suggested to affect the regulation of target gene mRNA by miRNAs. Limited studies have shown that miR-30a, miR-30e, and miR-34a are important in the pathogenesis of SCZ, and the miR-30 family is the predicted target of *COMT*. However, their association with the COMT gene is still a gap in the field of research. From this standpoint, we aimed to investigate whether the three miRNAs (miR-30a-5p, miR-30e-5p, and miR-34a-5p) may be involved in the targeting of the COMT gene.

## Materıals and Methods

### Bioinformatics Tools Used for miRNA–mRNA Target Prediction

Current online databases (Diana-MicroT, miRanda, PicTar, RNA22, TargetScan, miRDB, miRTarbase) for miRNA–mRNA interactions were used for evaluating possible COMT targets.

### Cellular Model of Schizophrenia

SH-SY5Y neuroblastoma cells are the most popular in vitro model used in neuropsychiatric research due to their dopaminergic and adrenergic properties. In this study, SH-SY5Y cells were cultured in Dulbecco’s modified Eagle’s medium (DMEM-low glucose, Capricorn) supplemented with 10% FBS (Gibco, USA), 1% penicillin/streptomycin (Gibco, USA), and 1% L-glutamine incubated at 37 °C in 5% CO_2_.

### miRNA Mimic Transfection

When SH-SY5Y cells reached 60–70% density, cells were plated in 6-well plate wells (1 × 10^5^ cells/well). Lipofectamine 2000 transfection reagent (Invitrogen, USA) was used to transfect cells with hsa-miR-30a-5p mimic, hsa-miR-30e-5p mimic, hsa-miR-34a-5p mimic, and their negative control (NC) mimics (A.B.T. Laboratory Industry, Turkey). The transfection was carried out at a concentration of 10 nM and 20 nM for both the sense and the antisense strands. After the 20-min incubation period, the cells were transfected using a drip method into the wells of the 6-well plate. The cells were incubated for 48 h prior to collection for RT-qPCR analysis. Sense and antisense sequences of each negative control mimic and miRNA mimics are given in Table 1.

**Table 1:** Oligonucleotide sequences (5′ → 3′) of NC and miRNA mimics

hsa-miR-30a-5p sense	TGTAAACATCCTCGACTGGAAG
hsa-miR-30a-5p antisense	CTTCCAGTCGAGGATGTTTACA
hsa-miR-30a-5p NC sense	TCACAACCTCCTAGAAAGAGTAGA
hsa-miR-30a-5p NC antisense	TACTCTTTCTAGGAGGTTGTGATT
hsa-miR-30e-5p sense	TGTAAACATCCTTGACTGGAAG
hsa-miR-30e-5p antisense	CTTCCAGTCAAGGATGTTTACA
hsa-miR-30e-5p NC sense	TCACAACCTCCTAGAAAGAGTAGA
hsa-miR-30e-5p NC antisense	TCTACTCTTTCTAGGAGGTTGTGA
hsa-miR-34a-5p sense	TGGCAGTGTCTTAGCTGGTTGT
hsa-miR-34a-5p antisense	ACAACCAGCTAAGACACTGCC
hsa-miR-34a-5p NC sense	GGTTCGTACGTACACTGTTCA
hsa-miR-34a-5p NC antisense	TGAACAGTGTACGTACGAACC

### RNA Extraction and qRT-PCR Analysis

Total RNA was isolated to evaluate transfection efficiency using TRizol LS Reagent (Invitrogen, USA) according to the manufacturer’s instructions. RNA concentration was quantified using a NanoDrop ND-100 spectrophotometer (NanoDrop Technologies, USA). Total RNA is extracted from cell lysates and RNA immunoprecipitation samples. For miRNA quantification analysis, A.B.T miRNA cDNA synthesis kit and 2 × qPCR master mix are used.

For the quantification of COMT from cell lysates and RNA-IP samples, cDNA was synthesized from total RNA using iScriptTM cDNA Synthesis kit (BioRAD). qRT-PCR was performed using the iTaq 2XSYBR Mix kit (BioRAD). β-tubulin was used as an internal control. The primer pairs of mRNAs used in the current study were as follows: COMT (forward, 5′-TGGACGCCGTGATTCAGGAG-3′; reverse, 5′-GCCAGCGAAATCCACCATCC-3′) and β-tubulin (forward, 5′-GGTAACCAAATCGGTGCTGCTTTC-3′; reverse, 5′ ACCCTCAGTGTGTGACCCT-3′).

Relative changes in *COMT* gene expressions were determined using the comparative threshold conversion (2^−ΔΔCt^) method.

### Western Blotting

Pellets correspond to SH-SY5Y cells transfected with miRNA mimics and negative control mimics were lysed with ClearBand RIPA buffer (EcoTech Biotechnology). Protein concentrations were determined using the Bradford Protein Assay (Bioshop). A total of 50 µg of protein was analyzed by 10% SDS–polyacrylamide gel electrophoresis (SDS-PAGE; Bio-RAD) and transferred onto the polyvinylidene fluoride (PVDF) membrane using the Trans-Blot Turbo transfer system (Bio-Rad, USA). The resolved proteins were transferred to membranes and blocked with 5% nonfat milk (BioShop) for 1 h at room temperature. Membranes were incubated with antibodies against COMT (1:500; FineTest) and GAPDH (1:2000; CloudClone) at 4 ^°^C for 2 h. Membranes underwent TBST washing and were then incubated with an anti-rabbit secondary antibody (Booster, 1:1000). The Odyssey® Fc Imaging System (LI-COR Biosciences) was used for capturing the protein band images. Quantitative analysis of target protein bands calculated according to the gray value ratio of the target band to GAPDH bands.

### RNA Immunoprecipitation Assay (RNA-IP)

Dynabeads Protein G (Invitrogen, USA) was used to conduct RNA-IP experiments according to the manufacturer’s instructions. SHSY5 cells transfected with miRNA mimics and NC mimics were grown in T25 flasks and when they reached approximately 90% confluence lysed in NP40 buffer supplemented with protease inhibitor. Overall, 5 µg of each antibody was diluted in 200 µl PBS with 0.1% Tween 20. Then, magnetic beads were pre-incubated with rabbit anti-AGO2 (Finetest, China) and normal rabbit IgG (Cell Signaling, UK) antibodies for 10 min at 4 °C. Pre-cleared samples were added to Dynabeads-Ab complex and incubated for 2 h at 4 °C with a rotator. Dynabeads-Ab-Ag complexes were washed 5 times. Before RNA purification, beads were pelleted using a magnetic stand (Invitrogen, USA) and treated with proteinase K (Qiagen, Germany) for degrading the Argonaute proteins, disrupting the antibody binding, and eluting the RNA from the beads. Thereafter, the RNA was purified with a Trizol reagent (GeneAll, USA).

### Pathway Enrichment Analysis

DIANA-miRPath Tool v3.0 (https://dianalab.e-ce.uth.gr/html/mirpathv3/index.php?r=mirpath) was applied for conducting GO and KEGG pathway enrichment analyses of miRNAs selected in our study.

### Statistical Analysis

The graphs, calculations, and statistical analyses were performed using the GraphPad Prism software version 8.0.1 (GraphPad Software, USA). One-way ANOVA, two-way ANOVA, and unpaired Student’s *t*-test were used for comparisons of differential expressions of genes and cytotoxicity assessment. Statistical results with *p < 0.05, **p < 0.01, ***p < 0.001, or ****p < 0.0001 were considered statistically significant.

## Results

### Bioinformatics Tools Used for miRNA–mRNA Target Prediction

**Fig. 1:**
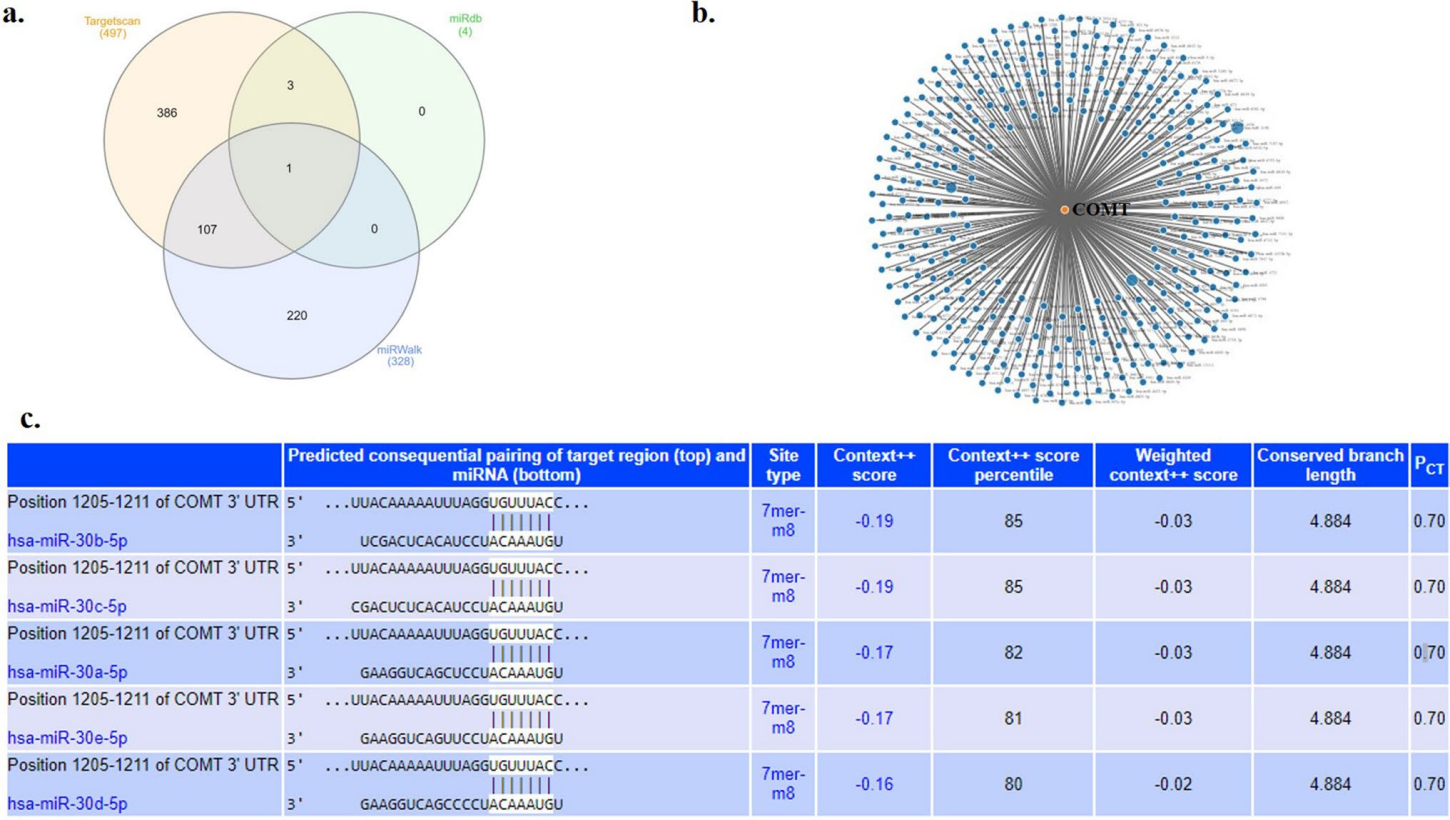
**a** Venn diagram of putative miRNA targeting to COMT using miRDB, miRWalk, and TargetScan target prediction software; **b** COMT network for predicted miRNAs using miRWalk software; and **c** miRNA-target complementarity of miR-30 family using TargetscanHuman database Release 8.0

Before performing high-throughput experiments, it is crucial to identify miRNA targets by computational methods. The complementarity between miRNA and target mRNA was the fundamental advantage for computational analysis. In order to identify potential miRNAs that directly regulate *COMT* expression, a thorough search was conducted on miRNA databases for putative binding sites in the 3′UTR of human *COMT* mRNA (Fig. 1a–b). Utilizing advancements in bioinformatic databases, we discovered that the miR-30 family is a potential target for *COMT* gene (Fig. 1c).

### Evaluation of miRNA Expression Levels After Mimic Transfections in SH-SY5Y Cells

**Fig. 2:**
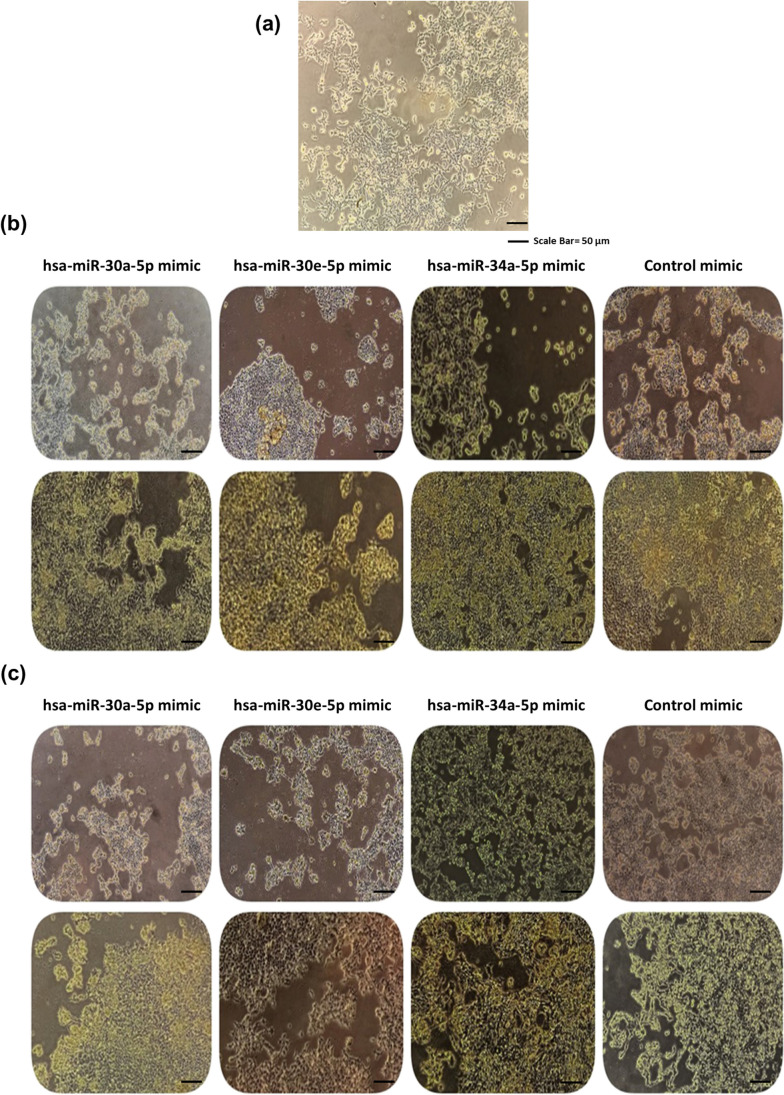
Microscopic images of SH-SY5Y cells (20 ×). **a** Typical cell morphology in the untreated control. **b** Microscopic images of SH-SY5Y cell morphology at 24 and 48 h at the stage of mimic transfection at 10 nM concentrations. **c** Microscopic images of SH-SY5Y cell morphology at 24 and 48 h at the stage of mimic transfection at 20 nM concentrations

**Fig. 3:**
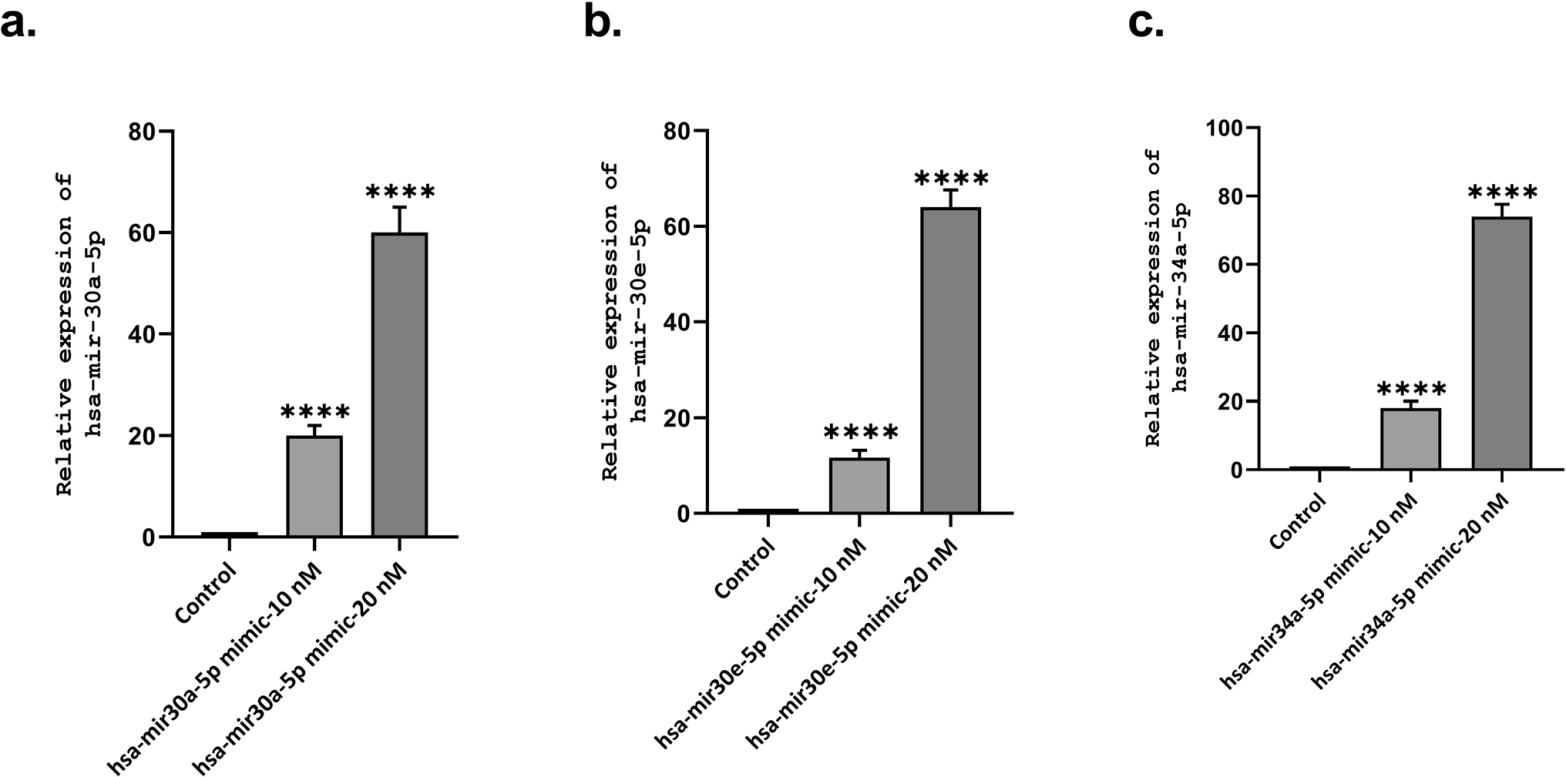
**a**–**c** miR-30a-5p, miR-30e-5p, miR-34a-5p, and their negative control mimics were transfected to SH-SY5Y cells using Lipofectamine 2000 reagent. Expression levels of miRNAs after miRNA mimic transfection were evaluated by RT-qPCR. Relative changes in the miRNA expressions were determined using the comparative threshold conversion (2^−ΔΔCt^) method. Statistical significance was determined using unpaired Student’s *t*-test (***p < 0.001; ****p < 0.0001)

SH-SY5Y neuroblastoma cells were transfected with hsa-miR-30a-5p mimic, hsa-miR-30e-5p, hsa-miR-34a-5p mimics, and their NC mimic oligonucleotides at concentrations of 10 nM and 20 nM for 48 h. No morphological changes were observed as a result of transfection (Fig. 2a–c). The expression levels of hsa-miR-30a-5p, hsa-miR-30e-5p, and hsa-miR-34a-5p increased significantly after transfection compared to their negative control mimics. Figure 3a–c displays the expressions of miRNAs at different concentrations of mimics.

### COMT Expressions Were Deregulated in SH-SY5Y Cells Following the miRNA Mimic Transfections

**Fig. 4:**
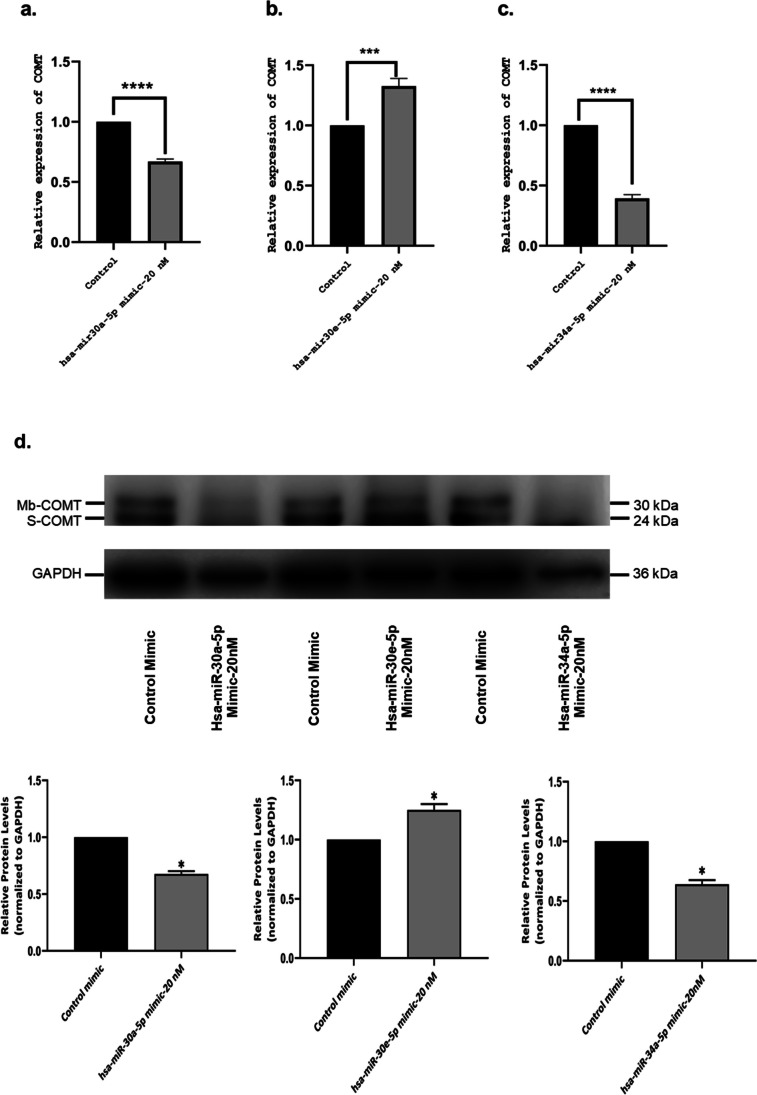
**a**–**c** COMT gene expression levels were evaluated after miRNA mimic transfection by RT-qPCR. **d** Western blot of COMT after miRNA mimic transfection. Statistical significance was determined using unpaired Student’s *t*-test (*p < 0.05; **p < 0.01; ***p < 0.001)

After the mimic transfection of each miRNA, the expression levels of COMT were evaluated as a function of the mimic transfection both on mRNA and protein levels. The qRT-PCR and western blot results indicate a decrease in the expression levels of *COMT* after hsa-miR-30a-5p and hsa-miR-34a-5p transfection and an increase following hsa-miR-30e-5p transfection compared to the samples transfected with negative control mimic (Fig. 4a–d).

### hsa-miR-30a-5p and hsa-miR-34a-5p Are Direct Targets for COMT Gene

Several miRNA databases were consulted for the identification of possible miRNAs that could be involved in the regulation of *COMT* expression. Three miRNAs, two belonging to the miR-30 family and miR-34a, were selected for the study. *COMT* is predicted to be a target of miR-30a-5p and miR-30e-5 as shown in Fig. 4, whereas there is no evidence in any database that miR-34a-5p is a target of *COMT*.

**Fig. 5:**
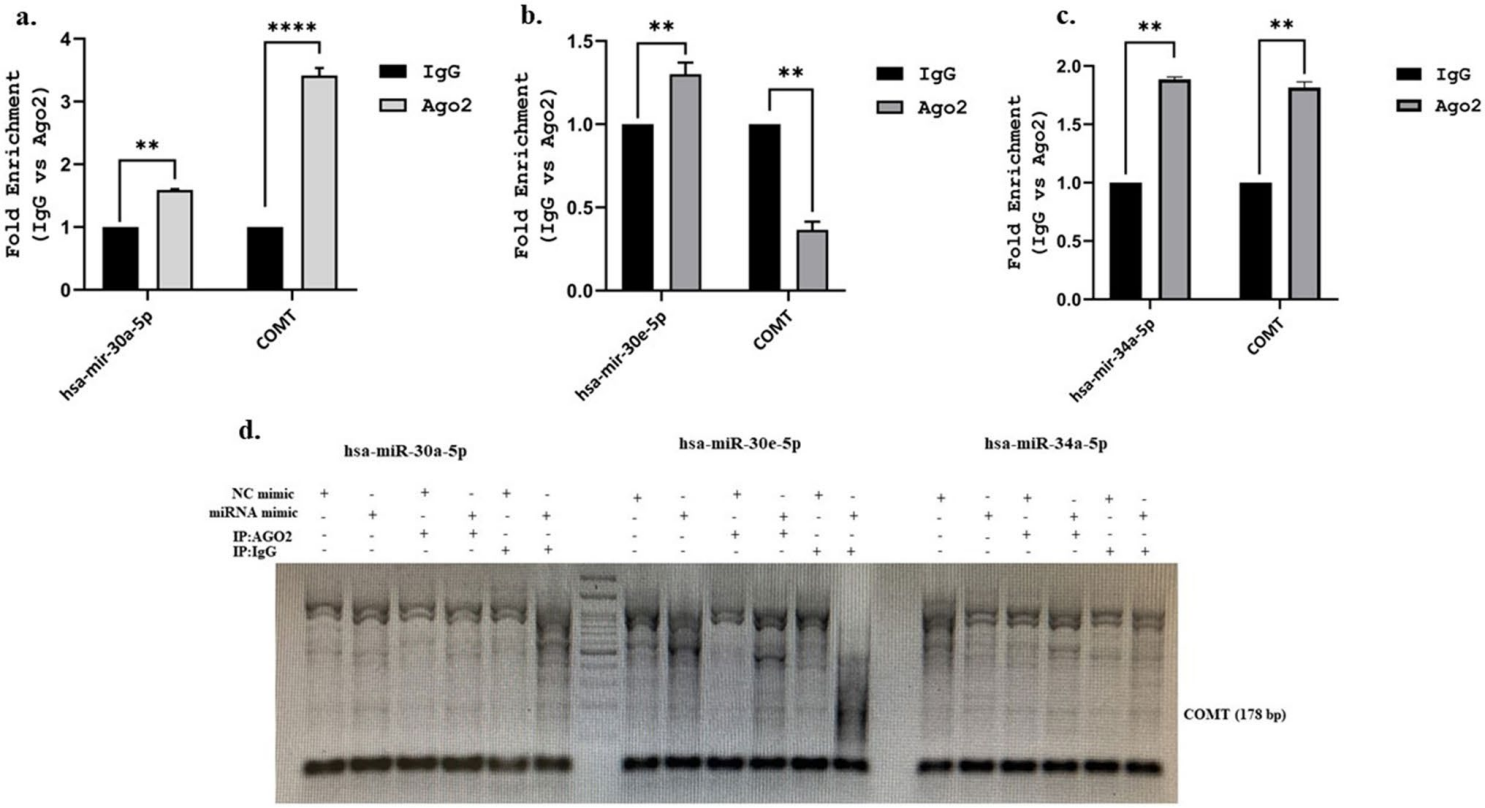
**a**–**c** The RNA-IP assay was performed to estimate the enrichment of COMT in SH-SY5Y cells transfected with miRNA mimics and negative control mimics. **d** RNA-IP samples were subjected to qPCR amplification using specific primer pairs for COMT. Statistical significance was determined using two-way ANOVA (**p < 0.01; ****p < 0.0001)

After transfections of mimics based on AGO2 enrichment of miRNA-bound targets on SH-SY5Y cell lysates, we performed RNA immunoprecipitation (RIP) experiments using an antibody against AGO2 to assess whether selected miRNAs bind to the *COMT* gene. RNA samples were prepared from cell lysates that were immunoprecipitated with Ago2 and IgG antibodies. The results showed that in SH-SY5Y cells transfected with selected miRNA mimics, the enrichment of the COMT transcript pulled down by AGO2 was increased after miR-30a-5p and miR-34a-5p compared to the negative control mimics by RNA-IP assay. These findings provide evidence that hsa-miR-30a-5p and hsa-miR-34a-5p associate with AGO2 protein to form an RNA-induced silencing complex (RISC) in SH-SY5Y cells (Fig. 5a–c).

Following washing and dissociation of miRNA mimic and NC mimic-transfected RNA-IP samples which were incubated with rabbit anti-Ago2 antibody and normal rabbit IgG, the samples were also subjected to RT-PCR with primers specific for *COMT.* Amplification products were assessed by electrophoresis on 1% agarose gels (Fig. 5d).

### Functional and Pathway Enrichment Analysis of Selected miRNAs

**Fig. 6:**
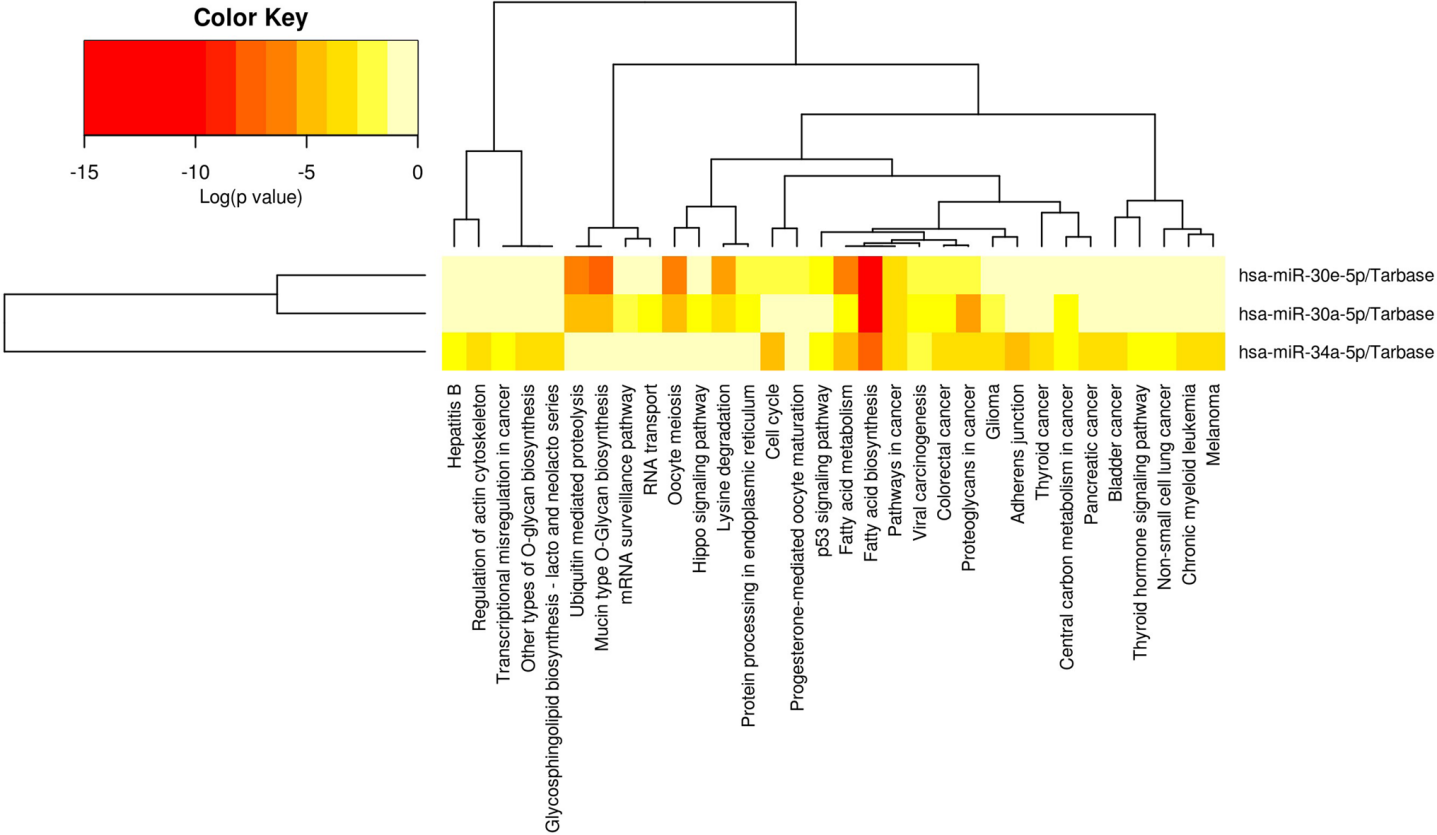
Heatmap of each miRNA (columns) and the target pathway (rows) interaction based on the significance (log *p*-value)

The DIANA-miRPath tool was utilized to pinpoint pathways that were enriched in genes that were considerably targeted by the three miRNAs of interest. The heatmap generated by Fisher’s exact test displays the significance (log *p*-value) of miRNA-pathway interactions (Fig. 6).

## Dıscussıon

The neurodevelopment of schizophrenia is associated with genetic and environmental factors that lead to inappropriate connections of neurons in the perinatal period. Multiple effects such as infections or psychosocial trauma play a role in the pathophysiology of schizophrenia [31, 32]. Dopamine is a potent neurotransmitter that governs neuronal functions in the central nervous system. Studies have shown that non-coding RNAs modulate nearly all aspects of dopamine signaling. Targeting of dopamine receptor signaling is driven by selected miRNAs, and miRNAs have emerging roles for both short and long non-coding RNAs in synaptic transmission [33]. In addition, miRNAs play a crucial role in neural development, differentiation, and maturation. Therefore, it is probable that the dysregulation of these pathways is associated with schizophrenia as it affects the cellular pathways involved in the expression of related genes [34]. *COMT* is a crucial target for disease treatment as it functions as a susceptibility gene for schizophrenia [35]. The aim of this study was the identification of a specific miRNA that has a crucial regulatory role for COMT in schizophrenia. In order to find a predicted miRNA, we searched all databases specific to miRNA–mRNA regulatory networks. It has been observed in the Targetscan database that positions 2 to 8 of the seed sequence of the miR-30 family and the 3′ UTR region of COMT exhibit an exact match (Fig. 1). Therefore, the relationship of two members of this family, miR-30a-5p and miR-30e-5p, with *COMT* was investigated, on the assumption that they could be direct targets for *COMT*.

The miR-30 family comprises five members and six mature miRNA sequences as miR-30a, miR-30b,miR-30c-1,miR-30c-2,miR-30d, and miR-30e. All members have a shared common seed sequence near the 5′ end. Nevertheless, they display distinct compensatory sequences near the 3′ end, thus enabling them to target various genes and pathways [36]. Based on this, we investigated whether 3 miRNAs, miR-30a-5p, miR-30e-5p, and miR-34a-5p, target *COMT* in our study.

miR-30a-5p is a miRNA molecule with a regulatory role in neuroprotection associated with central nervous system function. Little is known about whether osteogenic errors in BMSCs are related to the abnormal expression level of miR-30a-5p [37]. Since microRNAs have the potential to silence hundreds of genes involved in neuropsychiatric disorders, changes in miR-30a-5p are thought to be related to other gene regulation pathways [38]. Studies have shown that miR-30 family members are reduced in the prefrontal cortex of patients with schizophrenia when compared to healthy individuals, and miR-30b expression is significantly decreased in the cerebral cortex of schizophrenic patients [39]. A study investigating the effects of miRNAs indicated that miR-181b, miR-30e, miR-346, miR-34a, and miR-7 contribute significantly to the molecular mechanism of schizophrenia. It is becoming increasingly recognized that the aberrant expression of a number of miRNAs is important in the pathophysiology underlying schizophrenia. There are few studies elucidating the correlation between changes in miRNA expression and symptom amelioration in individuals with schizophrenia. When comparing the younger and older age groups, miR-30e expression was found to be significantly higher in the younger age groups. This suggests that miR-30e is the only miRNA that shows a differential expression in patients with schizophrenia at an early stage of the disease [21]. In addition, recent studies still lack information on the exact miRNA and their gene targets that mediate glial functions in schizophrenia [27]. Microarray analyses showed that 33 miRNAs, including miR-30d and miR-30e, were decreased in peripheral blood mononuclear cells isolated from schizophrenia patients. It has been reported that the biogenesis of 17 miRNAs is tightly controlled by a variety of regulators, among which are transcription factors that have an effect at the transcriptional level [39]. Multiple studies have associated abnormal expression of miR-30e-5p with schizophrenia. miR-30e-5p levels were increased in plasma, peripheral leukocytes, and peripheral blood mononuclear cells, as well as in the prefrontal cortex of patients with schizophrenia. How miR-30e-5p is linked to the pathophysiology of schizophrenia is unclear [40]. The levels of miRNA expression assessed in PBMC were compared between the schizophrenia and control groups. The findings revealed that in the schizophrenia group, miR-212, miR-34a, and miR-30e were notably up-regulated in comparison to the control group [21]. Furthermore, another study identified seven miRNAs (hsa-miR-34a, miR-449a, miR-564, miR-432, miR-548d, miR-572 and miR-652) as potential schizophrenia biomarkers [40]. Among these miRNAs, hsa-miR-34a was most altered in the PBMCs of schizophrenia patients. The significance of miR-34a-5p is highlighted by the fact that it has been shown to be elevated in the dorsolateral prefrontal cortex and plasma of schizophrenia patients [41]. However, miR-34a-5p was not identified as a possible target of *COMT* in database searches. We therefore investigated whether miR-34a-5p also targets *COMT*, given its importance in studies of schizophrenia patients.

In this study, we first investigated the effect of increased expression of selected 3 miRNAs on *COMT* expression in SH-SY5Y cells, which are used as a model for schizophrenia and we observed that higher levels of miR-30a-5p and miR-34a-5p inhibited COMT expression both on mRNA and protein levels (Fig. 4a–d). In our knowledge, miRNAs elicit their effect by silencing the expression of target genes. However, in a manner similar to RNAs, miRNAs may also function to regulate gene expression positively. We observed that higher levels of miR-30e-5p led to an elevation in the levels of *COMT*.

An interesting new biochemical approach to analyzing RISC-associated cellular mRNA has been reported in recent years. In human cells, AGO-immunoprecipitation (AGO-IP) has been associated with the overexpression of synthetic miRNAs. The use of AGO proteins can result in target genes that may not be physiologically relevant and may be subject to significant cell modulation. miRNAs bind members of the AGO protein family to form the RISC [42, 43] and methods based on AGO-IP have been used to reveal miRNA–mRNA network rearrangements [44]. Based on this, we performed RNA-IP to reveal the association of *COMT* with these predicted miRNAs in our study. Our data revealed that the enrichment of the COMT transcript pulled down by AGO2 was increased after miR-30a-5p and miR-34a-5p transfection providing important data that they could be binding directly to *COMT*. To date, no functional study has emerged for the association of miR-30a-5p and miR-34a-5p with *COMT*.

## Conclusıon

No studies were discovered in the literature regarding the impact of miR-30a-5p, miR-30e-5p, and miR-34a-5p on the regulation of the *COMT* gene. Further research is necessary to demonstrate the binding of COMT-miRNA on a molecular level, but we consider our findings on the impact of these miRNAs on the *COMT* as a potential point of reference for future research.

## Data Availability

All data supporting the findings of this study are available in the paper.
