# Correction: An In Vitro Study for the Role of Schizophrenia-Related Potential miRNAs in the Regulation of *COMT* Gene

**DOI:** 10.1007/s12035-024-04112-9

**Published:** 2024-03-19

**Authors:** Onur Tonk, Pervin Elvan Tokgun, Özge Sarıca Yılmaz, Onur Tokgun, Kubilay Inci, Büşra Çelikkaya, Nuray Altintas

**Affiliations:** 1 Faculty of Medicine, Department of Medical Biology, Celal University, Manisa, Turkey; 2 Faculty of Medicine, Department of Medical Genetics, Pamukkale University, Kınıklı, Denizli Turkey; 3 Department of Cancer Molecular Biology, Institute of Health Sciences, Pamukkale University, Denizli, Turkey

**Correction: Molecular Neurobiology**

10.1007/s12035-024-04070-2

The original version of this article unfortunately contained incorrect order of affiliations and author’s affiliation assignments as two affiliations; “Celal University Faculty of Medicine, Department of Medical Biology, Manisa, Turkey” and “Pamukkale University Department of Cancer Molecular Biology, Institute of Health Sciences, Denizli, Turkey” were missed.

The previous Affiliation 1 (Department of Medical Genetics, Pamukkale University) should now be affiliation 2, thus all authors should be now assigned to Affiliation 2.

The author Onur Tokgun will assigned to both 2 and 3.

The order of affiliations and authors's affiliation assignment should be corrected as follows:

Onur TONK^1^, Pervin Elvan TOKGUN^2^, Özge Sarıca Yılmaz^1^, Onur TOKGUN^2,3^, Kubilay INCI^3^, Büşra ÇELIKKAYA^3^, Nuray ALTINTAS^1^

1 Faculty of Medicine, Department of Medical Biology, Celal University, Manisa, Turkey

2 Faculty of Medicine, Department of Medical Genetics, Pamukkale University, K?n?kl?, Denizli, Turkey

3 Department of Cancer Molecular Biology, Institute of Health Sciences, Pamukkale University, Denizli, Turkey

The original article has been corrected.

